# Refining an Oral Health Care Partner Intervention Using Behavior Change Techniques

**DOI:** 10.1093/geroni/igab046.1599

**Published:** 2021-12-17

**Authors:** Ashley Leak Bryant, Rachel Hirschey, Courtney Caiola, Ya-Ning Chan, Brenda Plassman, Bei Wu, Donald Bailey, Ruth Anderson

**Affiliations:** 1 School of Nursing, Durham, North Carolina, United States; 2 UNC School of Nursing, Chapel Hill, North Carolina, United States; 3 East Carolina University, Greenville, North Carolina, United States; 4 UNC Chapel Hill, Chapel Hill, North Carolina, United States; 5 Duke University, Durham, North Carolina, United States; 6 New York University, New York, New York, United States; 7 duke University School of Nursing, Durham, North Carolina, United States; 8 University of North Carolina at Chapel Hill, UNC Chapel Hill, North Carolina, United States

## Abstract

Following a pilot, we refined an oral health carepartner intervention for individuals with mild dementia (IMD). In this intervention, we use behavior change techniques (BCTs) to foster changes by carepartners including using new oral-care techniques and developing skills for using cueing and communications approaches to support behavior changes by IMD (duration and frequency of toothbrushing and oral-hygiene skills); thus, improving plaque and gingival indices. We describe our approach to refining the intervention manual including a) completing the self-paced BCT taxonomy course, b) developing a coding schema, c) coding the original manual for evidence of BCTs, and d) refining the manual to improve use of BCTS in the refined intervention. Our results detail how BCTs can be applied to refine and improve interventions. This research demonstrates the value in using BCTs for interventions to address how carepartners and IMD can collaborate to improve oral hygiene care.

